# Carrier-free nano-integrated strategy for synergetic cancer anti-angiogenic therapy and phototherapy†
†Electronic supplementary information (ESI) available. See DOI: 10.1039/c8sc04123g


**DOI:** 10.1039/c8sc04123g

**Published:** 2019-01-09

**Authors:** Zheng Wei, Pingping Liang, Junqi Xie, Chuanhui Song, Chuanchao Tang, Yufeng Wang, Xiteng Yin, Yu Cai, Wei Han, Xiaochen Dong

**Affiliations:** a Central Laboratory of Stomatology , Nanjing Stomatological Hospital , Medical School of Nanjing University , 30 Zhongyang Road , Nanjing , 210008 , China . Email: iamycai@163.com; b Department of Oral and Maxillofacial Surgery , Nanjing Stomatological Hospital , Medical School of Nanjing University , 30 Zhongyang Road , Nanjing , 210008 , China . Email: doctorhanwei@hotmail.com; c Key Laboratory of Flexible Electronics (KLOFE) , Institute of Advanced Materials (IAM) , Nanjing Tech University (NanjingTech) , 30 South Puzhu Road , Nanjing , 211800 , China . Email: iamxcdong@njtech.edu.cn; d Pediatric Dentistry , Nanjing Stomatology hospital , Medical school of Nanjing University , 30 zhongyang road , Nanjing , 210008 , China

## Abstract

Herein, a nano-integrated strategy was used to combine an anti-angiogenic agent sorafenib and a photosensitizer chlorin e6 to form carrier-free multifunctional nanoparticles (SC NPs) for synergetic anti-angiogenic therapy and phototherapy. SC NPs (diameter, ∼152 nm) presented excellent water dispersity and passive targeting ability towards tumor sites *in vivo* based on the enhanced permeability and retention (EPR) effect, which could be monitored by fluorescence imaging. Besides, SC NPs exhibited effective reactive oxygen species (ROS) generation and photothermal conversion abilities for both photodynamic therapy (PDT) and photothermal therapy (PTT). At a rather low dosage (200 μg kg^–1^) and illumination with laser (660 nm, 500 mW cm^–2^), SC NPs could attack tumor tissues by killing the internal tumor cells *via* mild phototherapy, simultaneously cutting off the external nutrient and oxygen supplements of the tumor cells *via* anti-angiogenesis. Besides, oxygen consumption in the PDT process may be combined with anti-angiogenic therapy to further cause cell apoptosis by tumor starvation. In addition to the highly efficient therapeutic effect *in vivo*, SC NPs possessed excellent biosafety and biocompatibility, making them promising for fluorescence imaging-guided synergetic anti-angiogenic therapy and phototherapy in clinic.

## Abstract 2

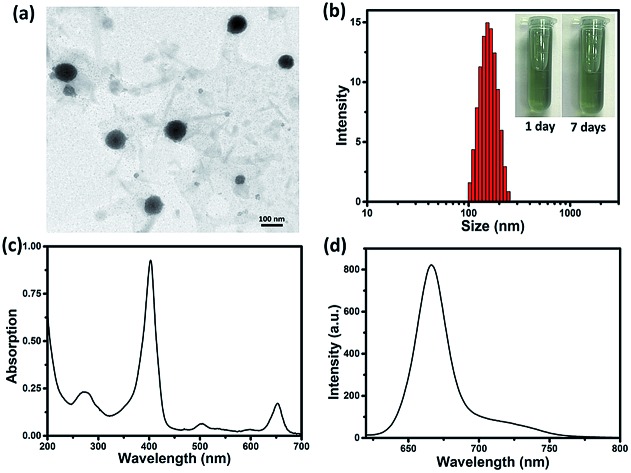
Herein, a nano-integrated strategy was used to combine an anti-angiogenic agent sorafenib and a photosensitizer chlorin e6 to form carrier-free multifunctional nanoparticles (SC NPs) for synergetic anti-angiogenic therapy and phototherapy.

## Introduction

Tumor blood vessels are oxygen and nutrient channels of the cancer cells and one of the main cancer transfer routines.^1^–^3^ As early as 1994, the Cancer Society of America recognized that the best way of cancer therapy is to cut off the lifeblood of tumors for the inhibition of tumor growth and stopping their transfer^4^–^6^ because any other way (surgery, radiation, chemotherapy, *etc.*) that directly kills the cancer cells only temporarily alleviates the disease, and it is difficult to avoid recurrence.^7^,^8^ Anti-angiogenic therapy as a relatively new cancer treatment method aims to suppress the expression of new blood vascular endothelial growth factor (VEGF) with tumor angiogenesis inhibitors and to induce the natural apoptosis of vascular endothelial cells for destroying the tumor angiogenesis net.^9^–^12^ By cutting off the blood supply, the rapid growth of tumors can be effectively inhibited on account of the blocked nutrients and oxygen. Therefore, the US Food and Drug Administration (FDA) has recommended that tumor angiogenesis inhibitors should be used as effective supplements along with surgery, radiation, and chemotherapy to prevent the tumor spread and metastasis.^13^,^14^ This strategy has significantly increased the five-year survival rate of cancer patients in America. However, most angiogenesis inhibitors, such as sorafenib,^15^ bevacizumab,^16^ pazopanib,^17^ are small organic molecules with inherent hydrophobicity and no tumor targeting ability. Consequently, ideal anti-angiogenic protocols and well-designed agents with water dispersity, targeting ability, and clinical prospects are extremely desired.

In the past decades, phototherapies,^18^–^22^ such as photodynamic therapy (PDT)^23^,^24^ and photothermal therapy (PTT),^25^–^27^ have attracted extensive research interests for targeting tumors due to the advantages of minimal harm to normal tissues, non-invasiveness, and efficient therapeutic ability.^28^–^30^ In PDT, photosensitizers (PSs) under light illumination can generate reactive oxygen species (ROS) including singlet oxygen (^1^O_2_) with internal oxygen to cause the tumor cell death, which also indicates that PSs may be effective deoxygenating agents in causing starvation of the tumor cells for apoptosis.^31^,^32^ Differently, PTT transforms light to heat with the aid of photothermal agents in the tumor to induce cellular hyperthermia-based necrosis or apoptosis.^33^,^34^ Nevertheless, PTT usually requires very high laser power (>1 W cm^–2^) that might cause damage to the surrounding normal tissues, while PDT efficacy is limited by the lesser oxygen levels in the tumor sites. Consequently, devising a synergistic tumor treatment to overcome the respective shortcomings and enhance the therapeutic efficiency is highly desirable.

Nanoparticles (NPs) hold great promise in selectively delivering therapeutic agents to tumors *via* the enhanced permeability and retention (EPR) effect, which is also well-known as passive targeting.^3^,^35^,^36^ Compared to small molecules, nano-agents have the ability to improve the therapeutic efficiency with a much lower dosage, which can decrease the side effects to the normal organs.^37^,^38^ Especially, organic dye-based nanoparticles show red-shift absorption and emission in the near infrared (NIR) window, which is beneficial to improve the deep penetration of bio-tissues for phototherapy.^39^,^40^ Moreover, the aggregated organic NPs may increase photothermal conversion under NIR irradiation for more efficient PTT.^41^–^44^ For instance, Hu *et al.* combined multifunctional polypyrrole (PPy), camptothecin (CPT), and thermo-cleavable doxorubicin (DOX) prodrug to form CPT@DOX/PPy NPs using a nano-integrated strategy, which was effectively applied for cancer photoacoustic imaging-guided photothermal-chemotherapy.^45^ Cook *et al.* designed metallacycles covalently bound to boron dipyrromethene (BODIPY) moieties to form self-assembled BODIPY-platinum supramolecular triangles for PDT and chemotherapy.^46^ However, these synergistic therapeutic nano-agents only focused on the tumor ignoring the fact that tumor cells still absorb nutrition from the surroundings with the intact blood vessels.

**Scheme 1:**
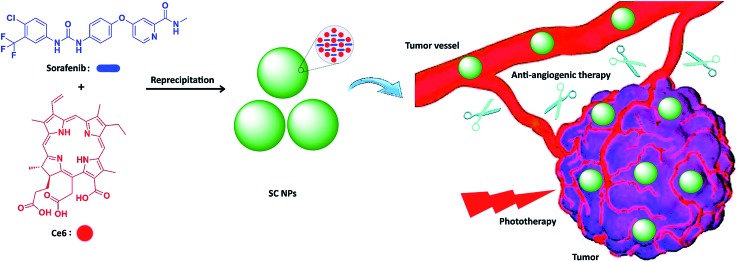
Schematic illustration of SC NP formation and their application for synergetic tumor anti-angiogenic therapy and phototherapy.

Herein, we integrated anti-angiogenic therapy and phototherapy (PDT and PTT) into one therapeutic strategy by assembling sorafenib (anti-angiogenic agent) and photosensitizer chlorin e6 (Ce6)^47^,^48^ into multifunctional nanoparticles (SC NPs) *via* the reprecipitation method. The resulting SC NPs presented good water dispersity and passive targeting ability towards tumors based on the EPR effect. These SC NPs could attack from outside and without the tumors by cutting off their external nutrient and oxygen supplements and simultaneously kill the internal tumor cells *via* PDT/PTT. Especially, due to the oxygen consumption in the PDT process, tumor cells may further starve, resulting in apoptosis, which collaborated with the anti-angiogenic therapy from the outside. Because of the synergistic effects from anti-angiogenic therapy and phototherapy, SC NPs could effectively cut off the tumor blood vessels and kill cancer cells simultaneously at a rather low dosage (200 μg kg^–1^) *in vivo* (Scheme 1).

## Results and discussion

### Synthesis and characterization

**Fig. 1:**
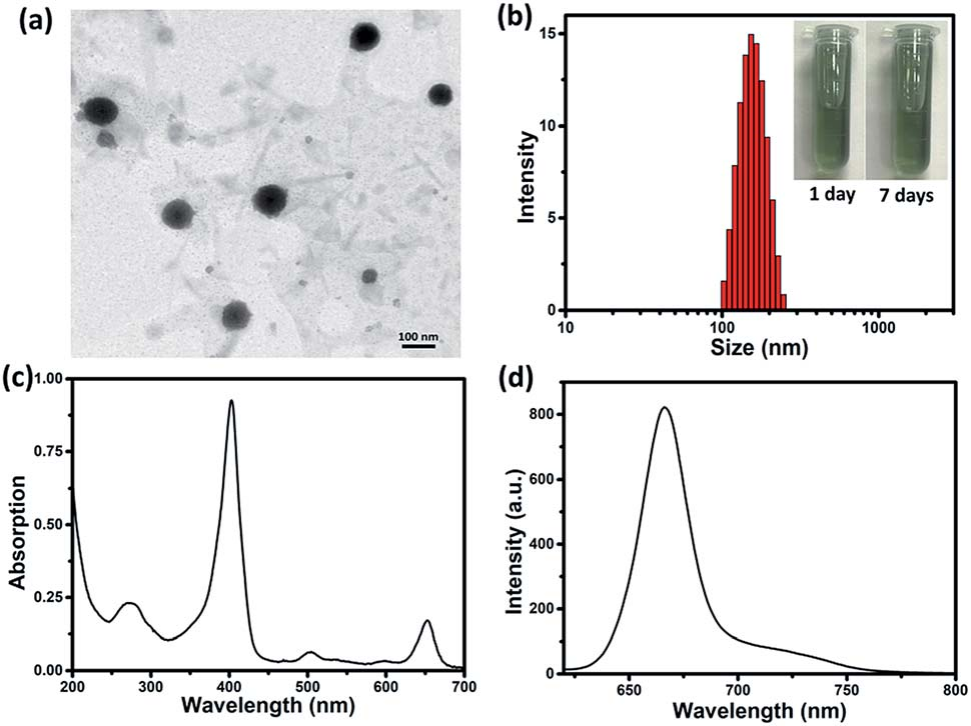
(a) TEM image of SC NPs. (b) DLS examination for size distribution of SC NPs in PBS (inset: photographs of SC NPs standing for 1 day and 7 days, 40 μg mL^–1^). (c) UV-Vis absorption spectrum of SC NPs in PBS (pH = 7.4). (d) Fluorescence emission spectrum of SC NPs in PBS (pH = 7.4).

By reprecipitation, hydrophobic sorafenib and Ce6 were converted into water-soluble SC NPs, which presented a dimension below 200 nm with spherical morphology, as observed by transmission electron microscopy (TEM, Fig. 1a). The dynamic light scattering (DLS) result showed that the mean size of the SC NPs was ∼152 nm (Fig. 1b), which resulted in suitable passive targeting ability towards tumors with the EPR effect.^49^ The phosphate buffer solution (PBS) of SC NPs with a turquoise color remained clear and transparent even after standing for 7 days under normal conditions, reflecting good stability. Besides, SC NPs presented excellent stability in plasma, indicating significant biological stability (Fig. S1†). The zeta potential value of SC NPs in water was measured as ∼31.99 mV, which further demonstrated their excellent stability. Due to the existence of Ce6, SC NPs demonstrated excellent NIR absorption in PBS with a peak at 654 nm (Fig. 1c), which was suitable for phototherapy *in vivo*. Moreover, as shown in Fig. 1d, the clear fluorescence peak of SC NPs in PBS emitted at 670 nm suggests their potential application in fluorescence imaging.

### *In vitro* multifunctionality

**Fig. 2:**
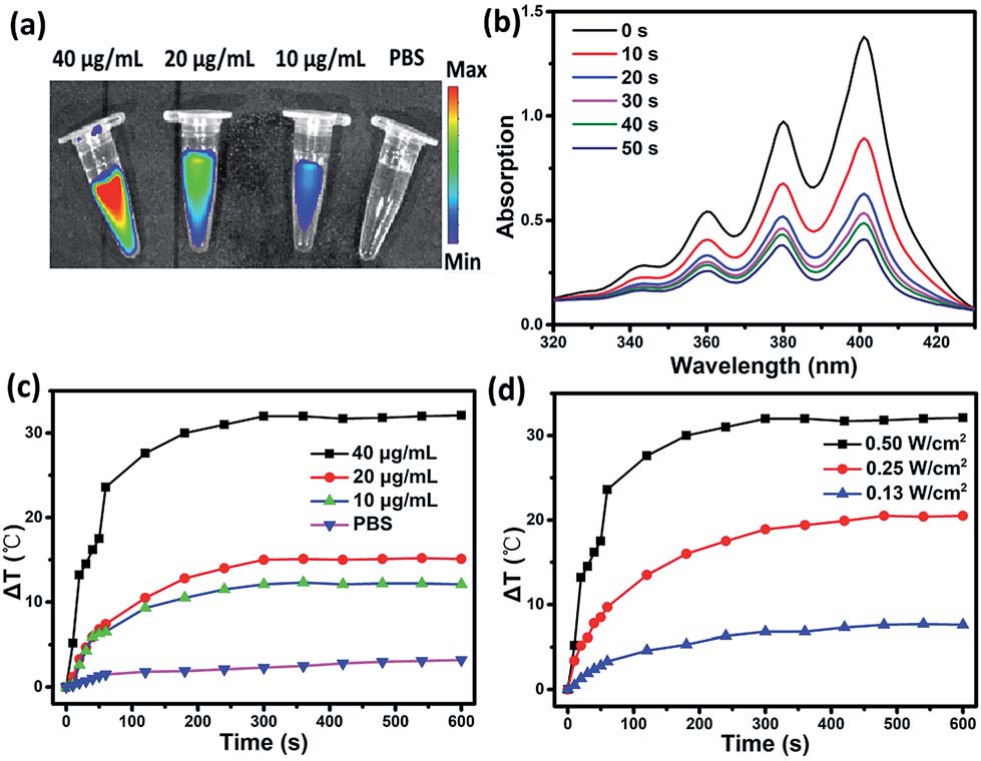
(a) The relationship between the fluorescence imaging intensity and the concentration of SC NPs in PBS (pH 7.4). (b) Absorption spectra at 379 nm of the mixture of SC NPs (40 μg mL^–1^) and ABDA (10^–4^ mol L^–1^) under illumination in PBS (pH 7.4) over time. (c) Photothermal conversion of SC NPs at different concentrations in PBS (pH 7.4) under laser irradiation (660 nm, 500 mW cm^–2^). (d) Photothermal conversion of SC NPs (40 μg mL^–1^) in PBS (pH 7.4) with different power densities.

To evaluate the multifunctionality of SC NPs, the photothermal conversion efficiency, fluorescence imaging property, and reactive oxygen species (ROS) generation of SC NPs were measured in PBS. As shown in Fig. 2a, the SC NP solution presented an apparent fluorescence signal, which was enhanced with the increase in concentration, and the fluorescence imaging intensity presented the brightest signal at 40 μg mL^–1^. Ce6 has been proven to be an excellent photosensitizer with efficient ROS generation; thereby, the ROS generation ability of SC NPs was further measured by using 9,10-anthracenediyl-bis(methylene)dimalonic acid (ABDA, absorption peak at 378 nm)^50^ as a probe. As shown in Fig. 2b, under the laser illumination (660 nm, 500 mW cm^–2^), the absorption density of ABDA at 378 nm rapidly decreased over time, indicating that SC NPs possess high ability to generate ROS in PBS for PDT. Fig. 2c shows the relationship between the temperature difference (Δ*T*) and SC NP concentrations under laser irradiation, showing the concentration dependence of the photothermal conversion. When the concentration of SC NPs increased to 40 μg mL^–1^, the temperature of the solution increased to 24 °C in 60 s. Then, Δ*T* gradually rose to over 30 °C. In the meantime, with the laser power increasing, Δ*T* of SC NP solution also increased (Fig. 2d). The photothermal conversion efficiency of SC NPs was calculated to be ∼48.0%, which was much higher than those of numerous other reported organic photothermal agents.^41^,^51^–^53^ These results indicate the multifunctionality of SC NPs as a new type of potential theranostic nano-agent for cancer fluorescence imaging, PDT, and PTT.

### Tumor cellular uptake and cytotoxicity

**Fig. 3:**
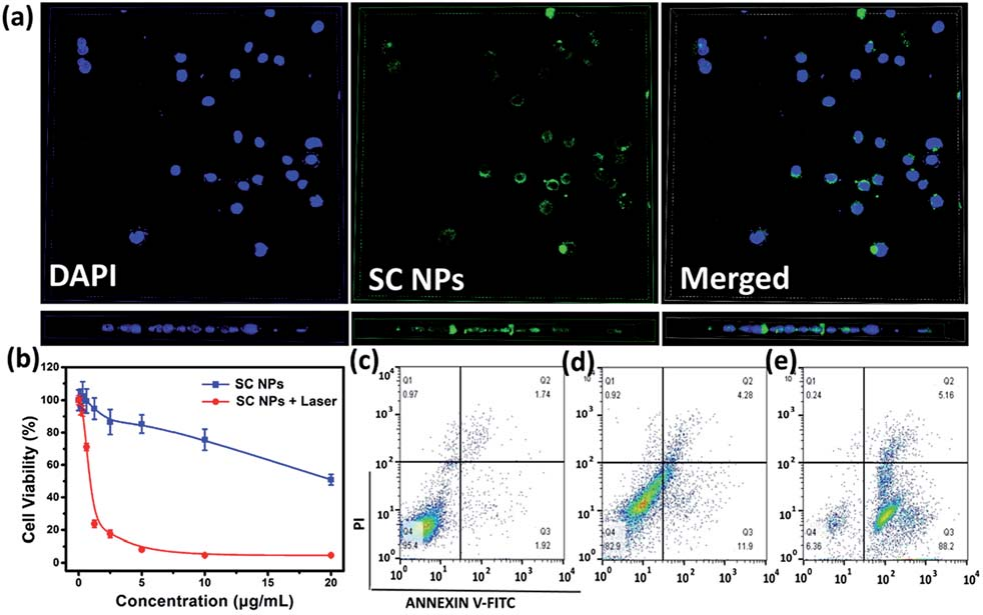
(a) Fluorescence imaging of SC NPs in HSC3 cells: left panel, DAPI channel; middle panel, fluorescence images; right panel, merged images; up panel, planform; down panel, side view. (b) CCK-8 assays of HSC3 cells incubated with SC NPs in different conditions. (c) Apoptotic rates of blank HSC3 cells. (d) Apoptotic rates of HSC3 cells treated with SC NPs only. (e) Apoptotic rates of HSC3 cells treated with SC NPs and laser irradiation.

SC NPs exhibited a fluorescence emission peak at ∼670 nm, indicating that the cellular uptake may be visualized. By the green color revelation of confocal fluorescence imaging, SC NPs (10 μg mL^–1^, 200 μL) could be readily taken up into HSC3 cells with incubation for 24 h (Fig. 3a, up panel). Moreover, by using confocal microscopy for 3D reconstruction of the specimen surface, SC NPs were confirmed to be located in the HSC3 cells *via* observations of the cell profile (Fig. 3a, down panel), and a video of the 3D view of the cells was recorded (ESI†). The cytotoxicity was assessed by using Cell Counting Kit-8 (CCK-8) assays^54^*in vitro* in the HSC3 cells incubated with SC NPs at different concentrations with or without illumination (660 nm, 500 mW cm^–2^). It was found that the cell viability decreased with laser illumination, of which the half-maximal inhibitory concentration (IC_50_) value was calculated to be 0.8808 μg mL^–1^, thus indicating highly efficient PDT/PTT and chemotherapy synergetic effect of SC NPs (Fig. 3b). In contrast to the laser illumination group, the other group (no special irradiation) exhibited much lower cell toxicity (IC_50_: ∼22.45 μg mL^–1^) because of the slight chemotherapeutic effect of sorafenib, demonstrating the desirable biocompatibility of SC NPs. Most anticancer drugs activate cell apoptosis to kill tumor cells; thus, the FITC-Annexin V/propidium iodide (PI) method^55^ was employed for the apoptosis analysis. The HSC3 cells were cultured with SC NPs with or without the condition of laser illumination (660 nm, 500 mW cm^–2^) at the concentration of 10 μg mL^–1^, and a group of blank cells was taken as control. As shown in the flow cytometry analysis, the ratios of the apoptotic cells were 16.18% and 93.36% in no irradiation and irradiation groups, respectively (Fig. 3c–e). In comparison with the no irradiation group, laser irradiation promoted a much higher apoptotic cell rate with the same dosage. These results further demonstrate that SC NPs have great therapeutic effect on tumor cells induced by both phototherapy and chemotherapy.

### ROS generation *in vitro*

**Fig. 4:**
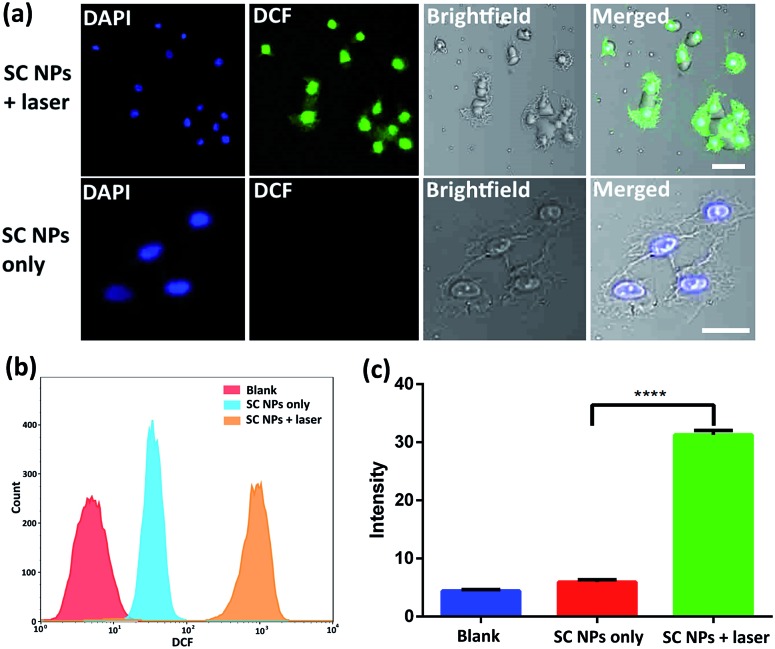
(a) Fluorescence imaging of SC NPs and DCFH-DA mixture in HSC3 cells with or without laser irradiation (660 nm, 500 mW cm^–2^). Scale bar: 50 μm. (b) Flow cytometric analyses. (c) DCF intensity in different groups. ****, *P* < 0.0001.

Cytotoxic ROS have been proven to cause cell damage in the process of PDT. The ROS generation ability of SC NPs was further examined by using 2′,7′-dichlorofluorescein diacetate (DCFH-DA) as a fluorogenic probe in the tumor cells. DCFH-DA has no fluorescence itself, and it produces 2′,7′-dichlorofluorescein (DCF) with fluorescence when reacting with ROS.^56^ As revealed in Fig. 4a, bright fluorescence imaging was viewed in the culture dish of SC NPs with DCFH-DA in HSC3 cells after laser irradiation (660 nm, 500 mW cm^–2^), which was caused by the generation of fluorescent DCF in HSC3 cells. In contrast, negligible fluorescence was emitted when these cells were kept out of light. The results of flow cytometry showed that the fluorescence amount in group of SC NPs with DCFH-DA in HSC3 cells under laser irradiation was more than an order of magnitude higher than those cells cultured with SC NPs without laser irradiation (Fig. 4b). By using a microplate reader, the DCF generation intensity of SC NPs with DCFH-DA in HSC3 cells under laser irradiation was further confirmed (Fig. 4c).

### Anti-angiogenesis assay *in vitro*

**Fig. 5:**
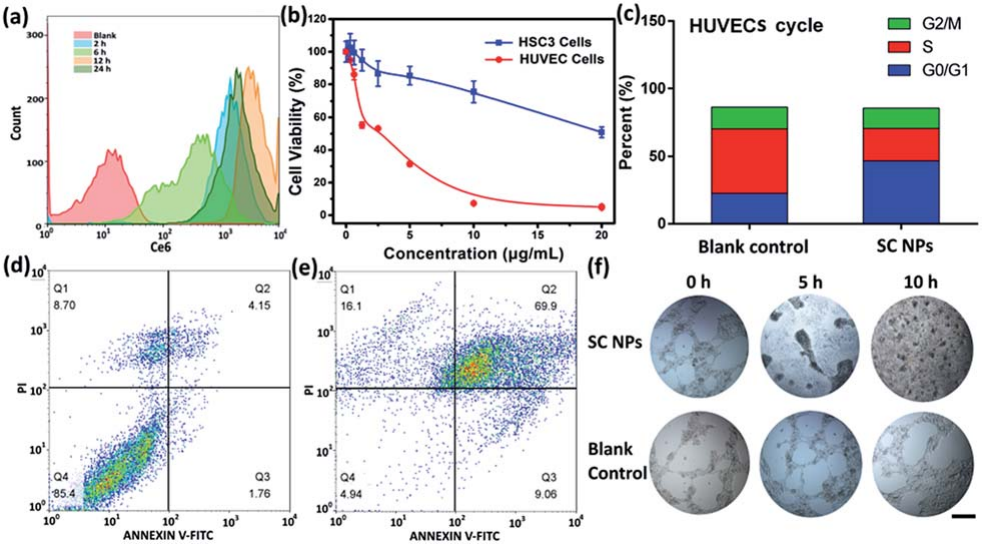
(a) Flow cytometric analyses of SC NP uptake in HUVECs. (b) CCK-8 assays of HSC3 cells and HUVECs incubated with SC NPs. (c) HUVEC cell cycle incubated with SC NPs. (d) Apoptosis of blank control HUVECs. (e) Apoptosis of HUVECs incubated with SC NPs. (f) *In vitro* endothelial cell tube formation and anti-angiogenesis assay. Scale bar: 100 μm.

To determine the anti-angiogenic effect of SC NPs in tumor endothelial cells, uptake, cytotoxicity, cell cycle, cell apoptosis, tube formation and anti-angiogenesis assays were assessed. By using the flow cytometric analyses, as shown in Fig. 5a, SC NPs (10 μg mL^–1^, 200 μL) could be gradually taken up into the human umbilical vein endothelial cells (HUVECs) after incubation for 12 h (ref. 57) and slightly discharged at 24 h, which indicated that SC NPs can be efficiently endocytosed into HUVECs. Sorafenib has been proven to have dual anti-tumor effects, which can not only be mediated by blocking the RAF/MEK/ERK signaling pathways and directly inhibiting the tumor cell proliferation, but also by inhibiting the expression of VEGF, platelet-derived growth factor (PDGF) receptor, and the formation of tumor angiogenesis to indirectly inhibit the growth of tumor cells.^58^ As shown in Fig. 5b, the cytotoxicity was assessed *in vitro* by using CCK-8 assays in HUVECs and HSC3 cells incubated with SC NPs of different concentrations. It can be found that the cell viability of HUVECs significantly decreased and IC_50_ was ∼0.2634 μg mL^–1^, indicating the high inhibitory effect of SC NPs on HUVECs. In comparison, the HSC3 cells exhibited much lower cell toxicity (IC_50_: ∼22.45 μg mL^–1^). Fig. 5c shows that the HUVECs treated with SC NPs were mostly arrested at the cycle *G*_0_/*G*_1_, indicating that the cells lost their ability to dissociate and turned to the process of differentiation, aging, and cell death. The FITC-Annexin V/PI method was futher employed for the death analysis of HUVECs incubated with SC NPs. As shown in Fig. 5d and e, in comparison with the blank control, HUVECs treated with SC NPs were promoted to reach a much higher apoptotic rate (79.0%), and the flow cytometry analysis showed that most HUVECs were apoptotic, which was induced by SC NPs. To further assess the anti-angiogenic effect of SC NPs, HUVEC cell tube formation and anti-angiogenesis assay was performed. As shown in Fig. 5f, after the cell tube formation was complete, SC NPs were added to these cell tubes, which gradually decomposed the fine structure of the blood vessels. At 10 h, almost no vessel structure was observed, indicating that SC NPs can effectively destroy the cell tubes *via* anti-angiogenesis. By contrast, the blood vessels in the blank control group presented the tendency to grow naturally.

### *In vivo* imaging

**Fig. 6:**
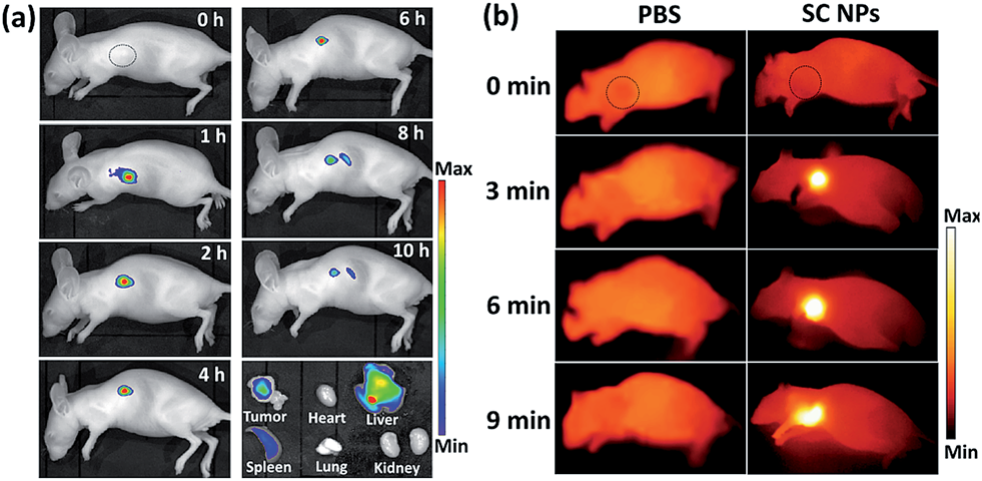
(a) *In vivo* fluorescence images of mice intravenously administrated with SC NPs (200 μg kg^–1^) at different time intervals. (b) *In vivo* infrared thermal images of mice intravenously administrated with PBS or SC NPs (200 μg kg^–1^) after 2 h under laser irradiation (660 nm, 500 mW cm^–2^) at different time intervals. Circles indicate the tumor site.

Fig. 6a shows the fluorescence images of mice with intravenous injection of SC NPs. After 1 h post injection, the fluorescence signal could be observed in the tumor sites and lasted for about 6 hours, indicating the excellent fluorescence imaging *in vivo* and EPR effect-based tumor targeting ability. The retention time of SC NPs in the tumor sites also provided abundant time for the subsequent treatment. After 8 h, the fluorescence decreased in the tumors, indicating that NPs were gradually eliminated from the tumors. After 10 h, these mice were sacrificed for *ex vivo* fluorescence imaging study. Weak fluorescence signal could be observed in the tumors as the SC NPs were wiped out from the tumor sites. Strong fluorescence was observed in the detoxification organ liver, which indicated that most of the SC NPs were metabolized by the liver. Based on the results of fluorescence imaging, *in vivo* thermal imaging of SC NPs was further investigated with laser irradiation (660 nm, 500 mW cm^–2^) after 1 h post injection. As revealed in Fig. 6b, the tumor temperature rapidly increases and reaches ∼55 °C with illumination for 6 min. In contrast, the tumor temperature of the control group showed nearly no change, which confirmed the good photothermal conversion of SC NPs and their passive targeting ability towards tumors.

### *In vivo* imaging-guided therapy

**Fig. 7:**
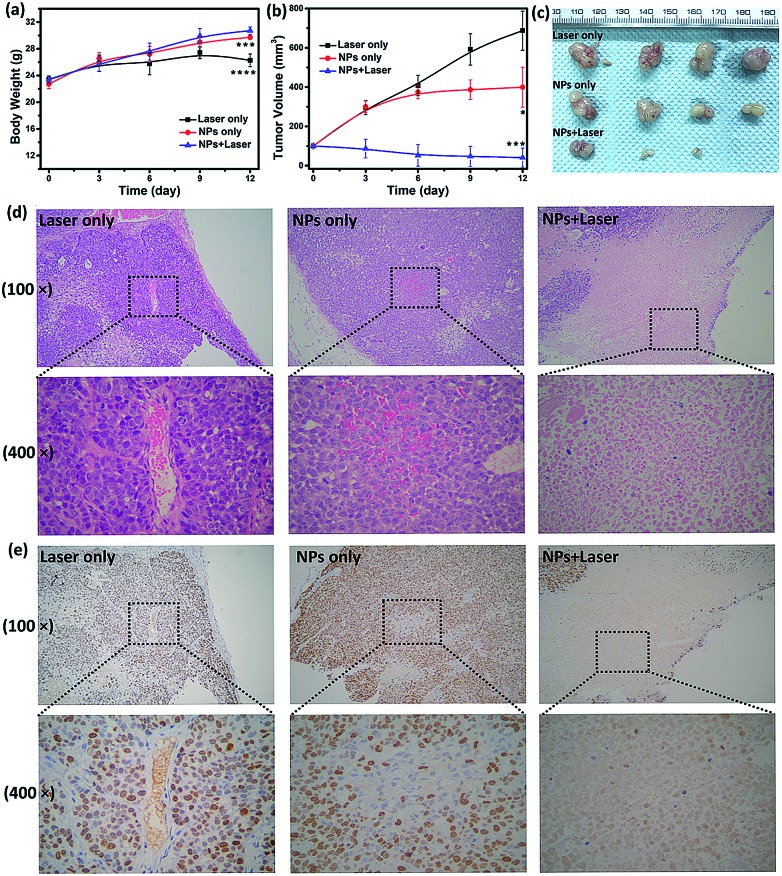
(a) Body weight change of the mice in different groups. (b) Changes of the tumor volume in different groups. *, *P* < 0.05; ***, *P* < 0.001; ****, *P* < 0.0001 (significant differences compared to the laser only group). (c) Tumors excised from the mice in different groups after the treatment for 12 days. (d) H&E and (e) Ki-67 staining of tumor tissues treated with laser only, SC NPs only, and SC NPs + laser groups.

To examine the *in vivo* toxicity and the therapeutic effect of SC NPs, HSC3 tumor-bearing mice were divided into 3 groups randomly for receiving different treatments. Fig. 7a shows that all groups exhibit similar increase in the body weight, indicating the low toxicity and good biocompatibility of NPs. As shown in Fig. 7b and c, the volume of tumors in the control group increased quickly, and the group injected with SC NPs without any special illumination presented slight tumor suppression, which may be caused by the anti-angiogenic effect of sorafenib in NPs. Remarkably, SC NP injection followed by laser illumination (660 nm, 500 mW cm^–2^) could greatly inhibit tumor growth. In contrast, free sorafenib (200 μg kg^–1^) and Ce6 (200 μg kg^–1^) were also injected in tumor-bearing mice with or without laser irradiation; both of the groups showed negligible therapeutic efficacy (Fig. S2†), indicating that the nanomedicines are much more effective than the traditional dosage form. All the mice were sacrificed at the end of 12 day treatments, and the haematoxylin and eosin (H&E) stained tumor tissue analyses^59^ were then conducted. Tumors in the control group presented very low differentiation of the tumor cells; the proportion of nucleolus was large and the morphology of cells was the same. Differently, necrotic cells could be found in the groups treated with either SC NPs or SC NPs + 660 nm laser irradiation (Fig. 7d). To further visually identify the proliferation of tumors, they were stained with the marker Ki-67.^60^ As shown in Fig. 7e, the tumor cells in the control group are dark brown and stained strongly positive; the number of positive cells was calculated to be over 80%, indicating that the cell proliferation was much stronger. On the contrary, tumors treated with SC NPs and laser irradiation showed negative Ki-67 staining, indicating that the proliferation of tumor cells was significantly weak. Besides, the positive cell number and Ki-67 intensity were only observed in the SC NP-treated groups. These examinations of tumor immunohistochemical study confirmed that the SC NPs + 660 nm laser irradiation group exhibited the highest inhibition efficacy and SC NPs have the ability to inhibit tumor growth.

**Fig. 8:**
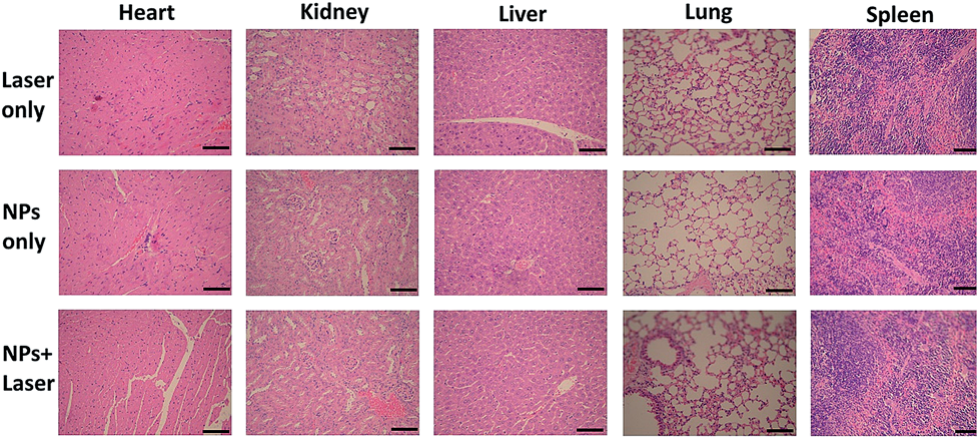
H&E staining images of major organs including heart, kidney, liver, lung, and spleen collected from HSC3 tumor bearing mice after treatment with laser only, SC NPs only, and SC NPs + laser groups, respectively. Scale bar: 100 μm.

The biosafety of SC NPs after treatment was also evaluated. As presented in Fig. 8, similar to the results for the blank control group, H&E staining results of the major organs showed no significant histological variations both in SC NPs only and SC NPs + laser irradiation groups, which indicated the considerable biosafety and biocompatibility of SC NPs.

## Conclusions

In summary, multifunctional carrier-free SC NPs were successfully prepared by the assembly of sorafenib and Ce6 *via* the reprecipitation method; they could attack from within and outside the tumor both by cutting off the external nutrient and oxygen supplements of tumor cells and killing the internal tumor cells *via* PDT/PTT. The SC NPs presented excellent water dispersity and passive targeting ability towards tumor tissues based on the EPR effect and could be monitored by fluorescence imaging *in vivo*. Due to the synergistic effect of anti-angiogenic therapy and phototherapy, SC NPs could effectively cut off the tumor blood vessels and kill cancer cells simultaneously with a rather low dose (200 μg kg^–1^) *in vivo*. Besides, the examinations of tumor immunohistochemical study confirmed that SC NPs exhibited high inhibition efficacy towards tumors and great biosafety and biocompatibility. We showed that SC NPs require much lower effective dose and moderate laser irradiation for synergistic anti-angiogenic therapy and phototherapy and have potential applications in clinic.

## Ethical statement

All animal procedures were performed in accordance with the Guidelines for Care and Use of Laboratory Animals of the Medical School of Nanjing University, and experiments were approved by the Animal Ethics Committee of Nanjing Stomatological Hospital.

## Conflicts of interest

The authors declare no conflict of interest.

## Supplementary Material

Supplementary information

Supplementary movie

## References

[cit1] Karimi A., Shahrooz R., Hobbenaghi R., Mohammadi R., Mortaz E. (2018). Cell J..

[cit2] Valko M., Leibfritz D., Moncol J., Cronin M. T. D., Mazur M., Telser J. (2007). Int. J. Biochem. Cell Biol..

[cit3] Wilhelm S., Tavares A. J., Dai Q., Ohta S., Audet J., Dvorak H. F., Chan W. C. W. (2016). Nat. Rev. Mater..

[cit4] Folkman J. (1995). Nat. Med..

[cit5] Gao M., Liang C., Song X., Chen Q., Jin Q., Wang C., Liu Z. (2017). Adv. Mater..

[cit6] Zhang C., Ni D., Liu Y., Yao H., Bu W., Shi J. (2017). Nat. Nanotechnol..

[cit7] Chen G., Jaskula-Sztul R., Esquibel C. R., Lou I., Zheng Q., Dammalapati A., Harrison A., Eliceiri K. W., Tang W., Chen H., Gong S. (2017). Adv. Funct. Mater..

[cit8] Sharma P., Allison J. P. (2015). Science.

[cit9] Zhang J., Li J., Shi Z., Yang Y., Xie X., Lee S. M., Wang Y., Leong K. W., Chen M. (2017). Acta Biomater..

[cit10] Carmeliet P., Jain R. K. (2000). Nature.

[cit11] Carmeliet P., Jain R. K. (2011). Nature.

[cit12] de Palma M., Biziato D., Petrova T. V. (2017). Nat. Rev. Cancer.

[cit13] Zhang Y., Chen Y., Zhang D., Wang L., Lu T., Jiao Y. (2018). J. Med. Chem..

[cit14] Roskoski Jr R. (2017). Pharmacol. Res..

[cit15] Zhao R., Li T., Zheng G., Jiang K., Fan L., Shao J. (2017). Biomaterials.

[cit16] Zhu R., Wang Z., Liang P., He X., Zhuang X., Huang R., Wang M., Wang Q., Qian Y., Wang S. (2017). Acta Biomater..

[cit17] Torok S., Rezeli M., Kelemen O., Vegvari A., Watanabe K., Sugihara Y., Tisza A., Marton T., Kovacs I., Tovari J., Laszlo V., Helbich T. H., Hegedus B., Klikovits T., Hoda M. A., Klepetko W., Paku S., Marko-Varga G., Dome B. (2017). Theranostics.

[cit18] Zhao H., Hu W., Ma H., Jiang R., Tang Y., Ji Y., Lu X., Hou B., Deng W., Huang W., Fan Q. (2017). Adv. Funct. Mater..

[cit19] Cheng L., Wang C., Feng L., Yang K., Liu Z. (2014). Chem. Rev..

[cit20] Lyu Y., Fang Y., Miao Q., Zhen X., Ding D., Pu K. (2016). ACS Nano.

[cit21] Zhu H., Fang Y., Miao Q., Qi X., Ding D., Chen P., Pu K. (2017). ACS Nano.

[cit22] Miao Q., Xie C., Zhen X., Lyu Y., Duan H., Liu X., Jokerst J. V., Pu K. (2017). Nat. Biotechnol..

[cit23] Liu J., Liang H., Li M., Luo Z., Zhang J., Guo X., Cai K. (2018). Biomaterials.

[cit24] Liu J.-n., Bu W., Shi J. (2017). Chem. Rev..

[cit25] Tang Z., Zhang H., Liu Y., Ni D., Zhang H., Zhang J., Yao Z., He M., Shi J., Bu W. (2017). Adv. Mater..

[cit26] Zhang Y., Yang D., Chen H., Lim W. Q., Phua F. S. Z., An G., Yang P., Zhao Y. (2018). Biomaterials.

[cit27] Lin H., Wang X., Yu L., Chen Y., Shi J. (2017). Nano Lett..

[cit28] Li J., Rao J., Pu K. (2018). Biomaterials.

[cit29] Jiang Y., Pu K. (2018). Acc. Chem. Res..

[cit30] Li J., Pu K. (2019). Chem. Soc. Rev..

[cit31] Cai Y., Tang Q., Wu X., Si W., Zhang Q., Huang W., Dong X. (2016). ACS Appl. Mater. Interfaces.

[cit32] Jia Q., Ge J., Liu W., Zheng X., Chen S., Wen Y., Zhang H., Wang P. (2018). Adv. Mater..

[cit33] Huang X., Zhang W., Guan G., Song G., Zou R., Hu J. (2017). Acc. Chem. Res..

[cit34] Yao X., Niu X., Ma K., Huang P., Grothe J., Kaskel S., Zhu Y. (2017). Small.

[cit35] Wang S., Shang L., Li L., Yu Y., Chi C., Wang K., Zhang J., Shi R., Shen H., Waterhouse G. I. N., Liu S., Tian J., Zhang T., Liu H. (2016). Adv. Mater..

[cit36] Sun T., Zhang Y. S., Pang B., Hyun D. C., Yang M., Xia Y. (2014). Angew. Chem., Int. Ed..

[cit37] Qi J., Sun C., Zebibula A., Zhang H., Kwok R. T. K., Zhao X., Xi W., Lam J. W. Y., Qian J., Tang B. Z. (2018). Adv. Mater..

[cit38] Qin W., Ding D., Liu J., Yuan W. Z., Hu Y., Liu B., Tang B. Z. (2012). Adv. Funct. Mater..

[cit39] Sun T., Dou J.-H., Liu S., Wang X., Zheng X., Wang Y., Pei J., Xie Z. (2018). ACS Appl. Mater. Interfaces.

[cit40] Zhu H., Li J., Qi X., Chen P., Pu K. (2018). Nano Lett..

[cit41] Cai Y., Si W., Tang Q., Liang P., Zhang C., Chen P., Zhang Q., Huang W., Dong X. (2017). Nano Res..

[cit42] Ding K., Zhang Y., Si W., Zhong X., Cai Y., Zou J., Shao J., Yang Z., Dong X. (2018). ACS Appl. Mater. Interfaces.

[cit43] Cai Y., Liang P., Si W., Zhao B., Shao J., Huang W., Zhang Y., Zhang Q., Dong X. (2018). Org. Chem. Front..

[cit44] Jiang Y., Cui D., Fang Y., Zhen X., Upputuri P. K., Pramanik M., Ding D., Pu K. (2017). Biomaterials.

[cit45] Yang J., Zhai S., Qin H., Yan H., Xing D., Hu X. (2018). Biomaterials.

[cit46] Zhou J., Zhang Y., Yu G., Crawley M. R., Fulong C. R. P., Friedman A. E., Sengupta S., Sun J., Li Q., Huang F., Cook T. R. (2018). J. Am. Chem. Soc..

[cit47] Xia F., Hou W., Liu Y., Wang W., Han Y., Yang M., Zhi X., Li C., Qi D., Li T., Martinez de la Fuente J., Zhang C., Song J., Cui D. (2018). Biomaterials.

[cit48] Hu T., He J., Zhang S., Mei X., Zhang W., Liang R., Wei M., Evans D. G., Duan X. (2018). Chem. Commun..

[cit49] Peng H. S., Chiu D. T. (2015). Chem. Soc. Rev..

[cit50] Cai Y., Tang Q., Wu X., Si W., Huang W., Zhang Q., Dong X. (2016). ChemistrySelect.

[cit51] Cai Y., Liang P., Tang Q., Yang X., Si W., Huang W., Zhang Q., Dong X. (2017). ACS Nano.

[cit52] Yoon H. J., Lee H. S., Lim J. Y., Park J. H. (2017). ACS Appl. Mater. Interfaces.

[cit53] Guo B., Sheng Z., Hu D., Li A., Xu S., Manghnani P. N., Liu C., Guo L., Zheng H., Liu B. (2017). ACS Nano.

[cit54] Duan X., Bai T., Du J., Kong J. (2018). J. Mater. Chem. B.

[cit55] Zhu X., Sun Y., Chen D., Li J., Dong X., Wang J., Chen H., Wang Y., Zhang F., Dai J., Pirraco R. P., Guo S., Marques A. P., Reis R. L., Li W. (2017). J. Controlled Release.

[cit56] Sun Q., You Q., Pang X., Tan X., Wang J., Liu L., Guo F., Tan F., Li N. (2017). Biomaterials.

[cit57] Song C., Zhang Y., Li C., Chen G., Kang X., Wang Q. (2016). Adv. Funct. Mater..

[cit58] Eldehna W. M., Fares M., Ibrahim H. S., Aly M. H., Zada S., Ali M. M., Abou-Seri S. M., Abdel-Aziz H. A., El Ella D. A. A. (2015). Eur. J. Med. Chem..

[cit59] Xue J., Zhao Z., Zhang L., Xue L., Shen S., Wen Y., Wei Z., Wang L., Kong L., Sun H., Ping Q., Mo R., Zhang C. (2017). Nat. Nanotechnol..

[cit60] Fluge O., Gravdal K., Carlsen E., Vonen B., Kjellevold K., Refsum S., Lilleng R., Eide T. J., Halvorsen T. B., Tveit K. M., Otte A. P., Akslen L. A., Dahl O., Norwegian Gastrointestinal Canc G. (2009). Br. J. Cancer.

