# Supplementary material for: Carrier-free nano-integrated strategy for synergetic cancer anti-angiogenic therapy and phototherapy
Source: Chem Sci. 2019 Jan 9;10(9):2778–84. doi: 10.1039/c8sc04123g (PMC6419942; doi:10.1039/c8sc04123g)
Supplement: Supplementary file 1 [file SC-010-C8SC04123G-s001.pdf]

Electronic Supplementary Material (ESI) for Chemical Science.
This journal is © The Royal Society of Chemistry 2019

# Supporting Information

## Carrier-Free Nano-Integrated Strategy for Synergetic Cancer Anti-angiogenic Therapy and Phototherapy

Zheng Wei[ab] ‡, Pingping Liang[c] ‡, Junqi Xie[ab], Chuanhui Song[ab], Chuanchao Tang[ab], Yufeng Wang[ab], Xiteng Yin[ab], Yu Cai* [ac], Wei Han* [ab], Xiaochen Dong* [c]

[a] Central Laboratory of Stomatology, Nanjing Stomatological Hospital, Medical School of Nanjing University, No 30 Zhongyang Road, 210008, Nanjing, China

[b] Department of Oral and Maxillofacial Surgery, Nanjing Stomatological Hospital, Medical School of Nanjing University, No 30 Zhongyang Road, 210008, Nanjing, China

[c] Key Laboratory of Flexible Electronics (KLOFE) & Institute of Advanced Materials (IAM), Nanjing Tech University (NanjingTech), 30 South Puzhu Road, 211800, Nanjing, China

‡These authors contributed equally

## Experimental Section

### Chemicals and Characterizations

Sorafenib, chlorin e6 (Ce6), 4', 6-diamidino-2- phenylindole (DAPI), 9, 10-anthracenediyl-bis (methylene) dimalonic acid (ABDA) and 2, 7-dichlorodihydrofluorescein diacetate (DCFH-DA) were buy from Adamas (China). Other solvents or reagents were used without further purified as received. Fluorescence emission spectra were measured on an F-7000 spectrometer (HITACHI, Japan). Absorption spectra were recorded on a UV-3600 UV-Vis-NIR spectrophotometer (Shimadzu, Japan). The size and Zeta potential of NPs were measured with a NanoPlus granulometer. The morphology of nanoparticles (NPs) was photographed on a transmission electron microscope (TEM, JEOL JEM-2100, Japan). The cell imaging was viewed by an Olympus IX 70 inverted microscope. Thermal images were photographed by an E50 infrared camera (FLIR, Arlington, VA).

### Ethical Statement

All animal procedures were performed in accordance with the Guidelines for Care and Use of Laboratory Animals of the Medical School of Nanjing University and Experiments were approved by the Animal Ethics Committee of Nanjing Stomatological Hospital.

### Preparation of SC NPs

200 μL of 2 mg/mL of sorafenib and 2 mg/mL Ce6 mixture in tetrahydrofuran solution was slowly added into 10 mL water at room temperature with vigorous stirring. Then, the mixture further stirred for 7 min, and the tetrahydrofuran in the solution was removed by nitrogen blowing. The NPs were achieved by centrifugation in the solution. The morphology and size of the NPs were determined by TEM and DLS, respectively.

### Singlet Oxygen Detection

ABDA was used to detect the singlet oxygen generation. SC NPs ($10^{-5}$ mol/L) were mixed with ABDA ($10^{-5}$ mol/L) in PBS (pH 7.4) at a dark room, and the absorption spectra of the mixture were immediately measured after irradiation with laser (660 nm, 500 mW/cm$^2$) over time.

***In Vitro* Photothermal Effect**

SC NPs in PBS (1 mL) were introduced into an eppendorf tube and irradiated with laser (660 nm), for 10 min. The temperature change was recorded by an E50 infrared camera.

**Cell Lines**

Human oral squamous cell carcinoma (HSC3) cells and Human umbilical vein endothelial cells (HUVECs) were purchased from the Cell Bank of the Chinese Academy of Sciences (Shanghai, China). Cells were cultured in Dulbecco's Modified Eagle's medium (DMEM, KeyGENE BioTECH, China) supplemented with 10% Fetal Bovine Serum (FBS, Gibco, USA), 1% streptomycin, and 1% penicillin, and maintained in a humidified incubator at 37 °C with 5% $CO_2$ for further measurements. Confluent cells were trypsinized with 0.05% trypsin (Gibco, USA) containing 0.02% ethylenediaminetetraacetic acid.

**Cellular Uptake Study**

The cellular uptake of HSC3 and HUVECs were visualized with a laser confocal scanning microscope (LCSM, NIKON, Japan) and Flow cytometer (Becton Dickinson, San Jose, CA, USA). HSC3 cells and HUVECs cells were seeded on a glass-bottomed culture dish at $3 \times 10^4$ cells/plate for visualized scanning and cells were seeded into 24-well plates at $3 \times 10^4$ cells/plate for flow cytometer study and cultured overnight. And then cells were incubated with DMEM containing 10 μg/mL of SC NPs for 2, 8, 10, and 24 h. For quantitative measurement, cells were washed three times by phosphate buffered saline (PBS), then cells

were collected and suspended with 1 mL PBS and the fluorescence intensity of the cells was excited at 660 nm (500 mW/cm$^2$) and detected by flow cytometer. The fluorescence histograms of SC NPs were recorded by flow cytometry. Images of cells were acquired by using a LCSM, the nuclei were stained with 4', 6-diamidino-2-phenylindole (DAPI).

**Detection of intracellular ROS production**

HSC3 cells were seeded into 6-well plates at $1 \times 10^5$ cells/well and cultured overnight. The experiment was divided into three groups (Group A: Blank control, Group B: SC NPs, Group C: SC NPs + 660 nm laser) in HSC3 cells. SC NPs was added at the concentration of 15 $\mu$g/mL and incubated for 24 h. To detect intracellular ROS production, the cells were incubated at 37 °C for 30min with 10 μL dichlorofluorescein diacetate (DCFH-DA, Beyotime, China) in the dark. Then the fluorescence emission was analyzed using LCSM, flow cytometry and spectrophotometry (SpectraMax M3, Molecular devices, Shanghai, China).

**Cytotoxicity Assay**

The cell cytotoxicity of SC NPs was measured using cell counting kit-8 (CCK-8, Biotool) in HSC3 and HUVECs cell lines. The experiment was divided into two groups (Group A: SC NPs, Group B: SC NPs + 660 nm laser) in HSC3 cells. HUVECs was treated with SC NPs. Cells were seeded into a 96-well plate at $5 \times 10^3$ cells/well and cultured overnight. Medium containing drugs were then added to each well, with SC NPs concentrations ranging from 0.03 to 40 μg/mL and incubated for an additional 24 h. Besides, cells were treated with 660 nm (500 mW/cm$^2$) laser for 5 min after the incubation. The cell viabilities in each group were determined using CCK8. Cells without any treatment were performed as negative control. The 50% of the cell growth inhibition ($IC_{50}$) was calculated by nonlinear regression analysis using GraphPad Prism 6 software (San Diego, CA).

**Tube Formation and Tube Broken Assay**

Tube formation assay of HUVECs was conducted on Matrigel (CORNING, USA). Matrigel was thawed at 4 °C overnight, spread evenly over each well (50 $\mu$L/well) of 96-well plates and polymerized for 30-45 min at 37 °C. HUVECs (3 × $10^4$ cells/well) were plated onto the matrigel layer and cultured in DMEM supplemented with 20% FBS. After 20 h of incubation at 37 °C, tube formation could be observed and captured with an inverted microscopy. Then SC NPs were added into the wells at the concentration of 15 $\mu$g/mL. After 5 h of incubation with SC NPs, the beginning tube broken could be observed. And at the incubation of 10 h, the tubes could be broken into clumps of different sizes. Pictures of broken tubes were captured with an inverted microscopy.

**Cell Apoptosis and Cell Cycle Assay by Flow Cytometry**

HSC3 and HUVECs were seeded into 6-well plates at 1 × $10^5$ cells/well and cultured overnight. The experiment was divided into three groups (Group A: Blank control, Group B: SC NPs only, Group C: SC NPs + 660 nm laser) in HSC3 cells. And the experiment was divided into two groups (Group A: Blank control; Group B: SC NPs) in HUVECs. SC NPs were added at the concentration of 15 $\mu$g/mL. After 24 h of incubation at 37 °C, cells were washed with PBS and conducted with Annexin V-FITC Apoptosis kit (Vazyme Biotech Co, Ltd, Nanjing, China). Cells were suspended with cold binding buffer. 5 μL Annexin V in 100 μL PBS were added for 10 minutes. Subsequently, 5 μL PI in 400 μL in was added. Finally, the entire mixture was incubated at 4 °C for another 15 minutes. In the part of cell cycle detection, Cells were suspended with PBS. 1 mL DNA Staining solution and 10 μL permeabilization solution were added and incubation for 30 min at room temperature. Cell cycle staining kit was purchased at MultiSciences (Lianke) Biotech Co., Ltd. All the

incubation was performed in the dark. Samples were detected with a flow cytometer (BD Biosciences) and the statics analyzed by FlowJo V10.

**Established Tumor-Bearing Mice Mode**

BALB/cJNJu-Foxn1nu/Nju male mice (4 weeks old) were purchased from Model Animal Research Center of Nanjing University. All animal experiments were conducted under the guideline of the Institutional Animal Care and Use Committee of the Medical School of Nanjing University. Mice were incubated in subcutaneous right forelimb armpit with the HSC3 cells at a density of 5-6 × $10^7$/ mL, 0.1 mL per mouse. Tumor volume was calculated as (tumor length) × $(\text{tumor width})^2/2$.

***In Vivo* Fluorescence Imaging**

100 $\mu$L of SC NPs (40 $\mu$g/mL) PBS solution was tail vein injected into HSC3 tumor-bearing mice. Fluorescence imaging of the tumors was monitored at different time and observed by a fluorescence imaging system (Bruker In-Vivo Imaging System Fx Pro.).

***In Vivo* Photothermal Imaging**

100 $\mu$L of SC NPs (40 $\mu$g/mL) PBS solution was tail vein injected into HSC3 tumor-bearing mice. After 1 h, photothermal imaging of the tumors was monitored at different time (0, 3, 6 and 9 minutes) by laser irradiation (660 nm, 500 mW/$cm^2$) and observed by an infrared camera.

***In Vivo* Tumor Treatment**

12 nude mice were injected HSC3 cells into the armpit as the tumor models. When the volumes of tumor grew to ~100 $mm^3$, these mice were randomly divided into 3 groups (Group A: Control, Group B: SC NPs only, Group C: SC NPs + 660 nm laser). Group A were tail vein injected with PBS every 2 days. Group B were tail vein injected with SC NPs PBS

(40 $\mu$g/mL, 100 $\mu$L) every 2 days, respectively. Group C were tail vein injected with SC NPs PBS (40 $\mu$g/mL, 100 $\mu$L) and irradiated by laser (660 nm, 500 mW/cm$^2$, 10 min) after injection for 1 h. This process above was carried out for 12 days, and the tumor size and body weight of the mice were measured every 2 days.

**Histology Examination**

The mice were sacrificed after the treatments for 12 days, after that, the histology analyses were conducted. Tumors and the major organs including livers, hearts, lungs, kidneys, spleens were taken from all the mice in different groups, and respectively fixed with 4% formaldehyde solution. The tissues were embedded in the paraffin cassettes after dehydration and stained with hematoxylin and eosin (H&E) or Ki-67. After that, the histology imaging was observed with a microscope.

**Statistical Analysis**

Data are expressed as the mean ± (standard deviation) SD. Statistical analysis was carried out using the Student's t test or one-way analysis of variance for multiple comparisons when appropriate. P values $< 0.05$ were regarded as significant. All the experiments were performed at least for three times.

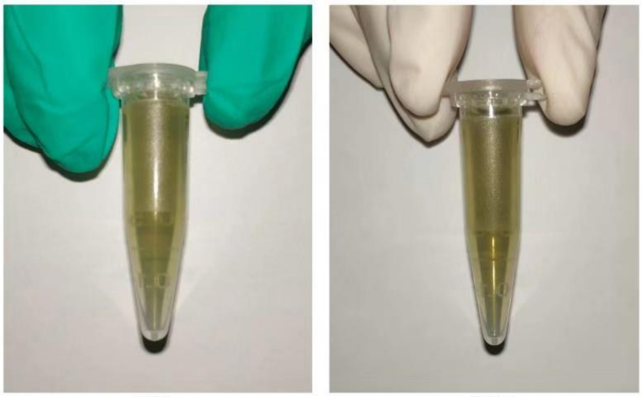


Figure S1. Photographs of SC NPs in plasma standing for 1 day and 7 days, 40 μg/mL.

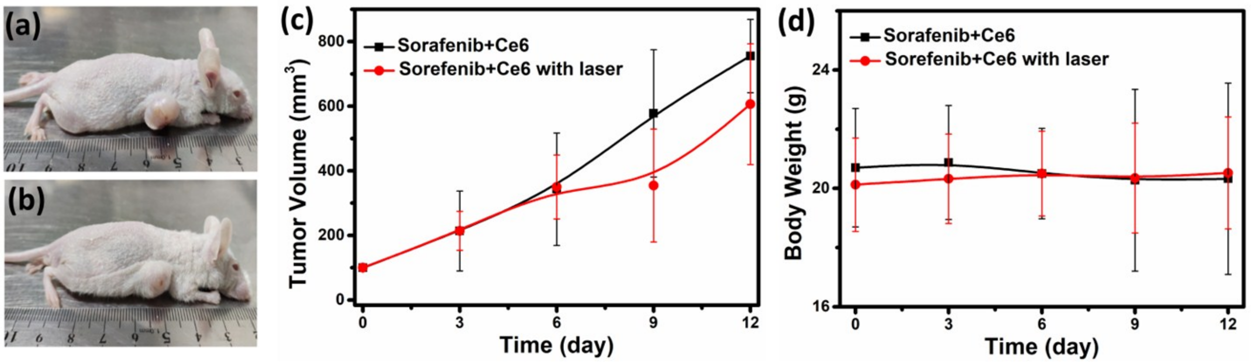


Figure S2. (a,b) Representative photographs of mice after 12 days treatment with sorafenib (200 μg/kg) and Ce6 (200 μg/kg) with/without laser irradiation. (c) Changes of the tumor volume in different groups. (d) Body weight change of the mice in different groups.
